# A Novel Swarm Intelligence-Driven Feature Selection for Interpretable Machine Learning in Multiparametric MRI-Based GBM Overall Survival Analysis

**DOI:** 10.3390/cancers18121888

**Published:** 2026-06-10

**Authors:** Abdulkerim Duman, Xianfang Sun, James R. Powell, Emiliano Spezi

**Affiliations:** 1 School of Engineering, Cardiff University, Cardiff CF24 3AA, UK; espezi@cardiff.ac.uk; 2 School of Computer Science and Informatics, Cardiff University, Cardiff CF24 4AG, UK; sunx2@cardiff.ac.uk; 3 Department of Oncology, Velindre University NHS Trust, Cardiff CF14 2TL, UK; james.powell2@wales.nhs.uk

**Keywords:** radiomics, GBM, brain tumor, survival analysis, quantitative imaging biomarkers, precision oncology, artificial intelligence

## Abstract

Background/Objectives: In this study, we develop and validate an interpretable machine learning (ML) model that integrates a hybrid swarm intelligence (SI)-based feature selection method with multiparametric magnetic resonance imaging (MRI)-derived RFs to estimate overall survival (OS) in glioblastoma multiforme (GBM) patients. Methods: A cohort of 276 GBM patients with open-access pre-treatment MRI data was used to perform comprehensive radiomic analysis. In the training (discovery) dataset, we employed five-fold cross-validation combined with bootstrapping to ensure robust methodological validation. Model evaluation covered the concordance index (C-index) with 95% confidence intervals (CIs). Additionally, survival stratification was performed using Kaplan–Meier curves and the log-rank test to separate patients into low- and high-risk groups for OS. The final survival model integrates patient age and ten independent RFs. Results: The model’s performance in the holdout test dataset was evaluated by a C-index of 0.71 (95% CI: 0.63–0.80), exhibiting statistically significant risk stratification (*p* = 3 × 10^−4^). Upon external validation, the model achieved a C-index of 0.67, maintaining statistical significance (*p* = 1 × 10^−2^). Conclusions: The research combined a traditional regularized Cox regression (Cox-LASSO) model with a new SI-based LASSO-PSO method, yielding significant stratification. To our knowledge, the present study offers one of the first studies to document the use of an interpretable ML model with an SI-based approach for successful risk stratification based on OS.

## Simple Summary

This study developed and validated an interpretable machine learning (ML) model using a novel swarm intelligence (SI)-based feature selection method with multiparametric magnetic resonance imaging (MRI)-derived radiomic features (RFs) to estimate overall survival (OS) in patients with glioblastoma multiforme (GBM). We used a cohort of 276 GBM patients and incorporated external validation from a different institution to ensure methodological robustness. The results are conducted with a limit of 10 features to enhance the interpretability of radiomic models. The model achieved good discriminatory performance (C-Index: 0.71) and significant patient stratification (*p* = 3 × 10^−4^) on the holdout test set. Additionally, it maintained comparable performance (C-Index: 0.67) and significant patient stratification (*p* = 1 × 10^−2^) on the external validation cohort. This study is, to our knowledge, among the first studies to integrate SI-driven feature selection and interpretable ML to achieve successful risk stratification in overall survival prediction.

## 1. Introduction

Glioblastoma multiforme (GBM) is the most common primary brain tumor in adults, described by its rapid growth, aggressive nature, and significant infiltration of adjacent brain tissue [1,2]. The World Health Organization (WHO) classifies GBM as a grade 4 malignancy of the central nervous system. This classification represents the highest grade possible, highlighting the tumor’s extremely aggressive nature and the poor prognosis [3]. The aggressive invasion and rapid growth of GBM hinders successful surgical treatment, complicating surgical removal and undermining the effectiveness of conventional therapies [4].

GBM is characterized by intrinsic genetic complexity and heterogeneity at both intertumoral and intratumoral levels, which present challenges in developing effective treatment options [4]. Tissue sampling through a surgical procedure with biopsy or resection is frequently required to diagnose GBM and is important for effective treatment planning and reliable prognostic assessment [5]. Complementing conventional biopsies, non-invasive imaging modalities offer essential insights for the comprehensive characterization of tumors and their sub-regions [5]. In contrast to conventional imaging modalities such as computed tomography (CT) and positron emission tomography (PET), magnetic resonance imaging (MRI) provides better tissue contrast resolution and avoids exposing patients to ionizing radiation [6].

As medical imaging analysis has progressed, radiomics has become a promising quantitative approach, allowing the extraction of computable features from medical images [7,8]. By providing quantitative insights into imaging patterns that are invisible to the human eye, radiomic analysis may enhance individualized treatment planning in GBM, enabling more precise and effective therapeutic interventions. Radiomics-based clinical decision-making may prioritize high interpretability over peak predictive performance for clinical trust [9]. Additionally, data limitations in rare diseases are an important challenge, as deep learning (DL) models are inefficient when trained on small datasets [10]. Therefore, traditional ML models might be preferable to deep learning models as they offer the intrinsic transparency [9] and maintain moderate performance on the limited datasets. For instance, Meneghetti et al. [11] adopted traditional approaches by utilizing two radiomic features (RFs), which yielded moderate predictive performance. Van Timmeren et al. [12] recommended that the incorporation of as many as 10 features can benefit the optimization of the model’s effectiveness, offering a distinct perspective on methodological design. Recent survival analyses in GBM integrated multiple clinical and RFs, although the inclusion of numerous features can limit reproducibility and interpretability [13,14,15].

Nature-inspired methods utilize randomness and an iterative search workflow to determine optimal feature subsets for feature selection. In radiomics, nature-inspired methods, most notably Genetic Algorithms (GAs) from evolutionary algorithms and particle swarm optimization (PSO) from swarm intelligence (SI) algorithms, are increasingly employed for feature selection, presenting promising solutions for analyzing high-dimensional data [16,17,18,19]. Hybrid methods that utilize nature-inspired algorithms might yield effective solutions for feature selection and predictive modeling, demonstrating competitive or superior performance while maintaining interpretability in applications related to GBM [16].

Traditional feature selection methods, such as LASSO, present computational efficiency [20] and small feature sets for model interpretability [21]. However, the method demonstrates limited reproducibility [22,23], the arbitrary exclusion of multicollinear variables [24], and ignoring nonlinear dependencies [25], resulting in a moderate impact on predictive performance in radiomic research [11,26].

Conversely, nature-inspired algorithms, such as Genetic Algorithms (GAs), provide good global search exploration [27]. On the other hand, particle swarm optimization (PSO) provides an effective trade-off between global and local search capabilities [28]. While GA is prone to information loss from previous iterations [17], PSO often suffers from radiomic feature redundancy and an inefficient random initialization strategy [19].

To address these limitations, we proposed a hybrid LASSO-PSO feature selection method that integrates linear efficiency with nonlinear exploration. Our approach employed the following three-step pipeline: (i) reducing multicollinearity via correlation analysis, (ii) utilizing LASSO with cross-validation to generate multiple enriched feature pools (ranging from two-feature to nine-feature), and (iii) applying PSO to perform a balanced search within these refined pools. By using LASSO to provide a pre-optimized initialization for PSO, this method handled feature redundancy. Additionally, the PSO-guided method captured nonlinear relationships, which enhanced predictive accuracy. It maintained robustness with a limited feature set in ML applications.

To address reproducibility challenges in radiomics-based GBM survival analysis, this study utilized widely used preprocessing and segmentation methods. The findings were also validated using open-access data from multiple institutions.

## 2. Materials and Methods

### 2.1. Study Population

This study employed a radiomic model development and validation framework based on 276 GBM cases, integrating data from the following two sources: 236 cases from the multi-institutional BraTS 2020 Challenge dataset [29,30,31], and 40 cases from the single-institutional Río Hortega University Hospital Glioblastoma Dataset (RHUH-GBM) [32]. In compliance with the Response Assessment in Neuro-Oncology (RANO) criteria [33], both datasets comprised the following four standardized preoperative MRI modalities: T1-weighted (T1), T1-weighted contrast-enhanced (T1ce), T2-weighted (T2), and fluid-attenuated inversion recovery (FLAIR). They also included information on overall survival (OS) and patient age.

### 2.2. Study Design

**Figure 1:**
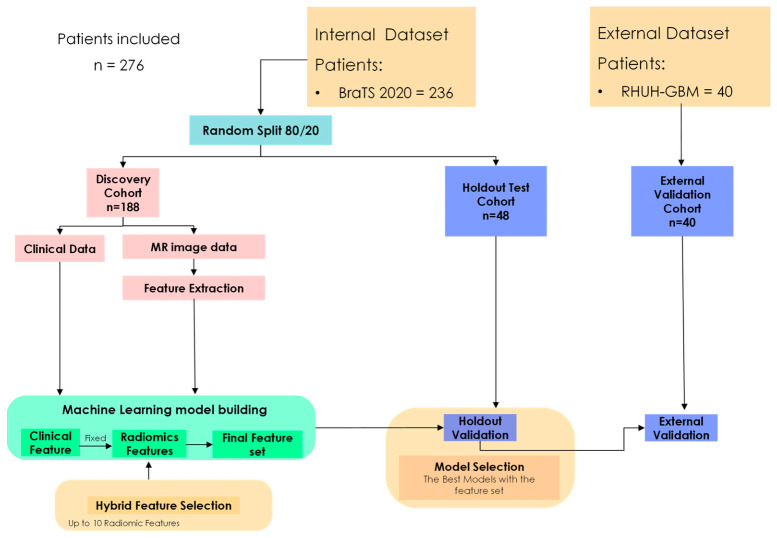
This study used an internal dataset, which consisted of a discovery cohort for selecting features and developing the model, along with a holdout test cohort for assessing performance on unseen data. Additionally, an external dataset was utilized to further validate the model’s generalizability.

The study, as shown in Figure 1, applied a time-to-event analysis for OS, stratifying cases into high- and low-risk groups. The endpoint was measured from the date of pathological diagnosis to death (censored = 1) or final follow-up (censored = 0). The BraTS 2020 dataset was divided into the following two subsets: a discovery cohort, representing 80% of the data (n = 188), utilized for model development, and an internal validation cohort representing 20% of the data (n = 48). Additionally, the RHUH-GBM dataset was employed as an external validation set to evaluate the generalizability of the model. Clinical parameters from the discovery cohort were combined with RFs extracted from the following three tumor regions: enhancing tumor (ET), tumor core (TC), and whole tumor (WT). Feature selection was performed using the discovery cohort, followed by the development and optimization of risk stratification models. Model performance was evaluated using the following two-step validation strategy: first, by testing on a holdout dataset, and second, by applying the models to an external validation cohort to assess their generalizability.

Risk stratification performance was assessed using the concordance index (C-index). Kaplan–Meier plots and log-rank tests were utilized to evaluate differences in survival outcomes across risk groups.

### 2.3. Image Pre-Processing and Feature Extraction

The BraTS dataset incorporates MRI scans from 19 institutions, representing a broad spectrum of imaging protocols and scanner settings. Preprocessing included DICOM-to-NIfTI conversion, followed by N4 bias field correction as a preliminary step for registration [34]. The spatial alignment procedure involved a series of steps as follows: first, aligning the T1, T2, and FLAIR sequences to the T1ce volume, and then registering them to the SRI24 atlas framework [35]. Consistent spatial sampling was achieved through isotropic voxel resampling ((1 × 1 × 1) mm^3^). A skull-stripping method was carried out using a pre-trained DL model. Z-score normalization was applied to mitigate scanner- and patient-specific intensity variations. Using CaPTk [36], the preprocessing pipeline included brain tissue extraction, intensity standardization, and spatial normalization, resulting in MRI volumes standardized to (1 × 1 × 1) mm^3^ voxel resolution and 240 × 240 × 155 matrix size.

To ensure compatibility with the BraTS 2020 dataset, the RHUH-GBM dataset was processed using a consistent preprocessing pipeline [32]. First, DICOM-to-NIfTI conversion was performed. Next, rigid registration was applied to align T1ce images to the SRI24 atlas, with the subsequent alignment of T1, T2, and FLAIR sequences to the transformed T1ce volume. Brain extraction was then performed on all registered sequences using a DL-based method. Finally, Z-score intensity normalization was applied to all images. Uniformity in voxel resolution of (1 × 1 × 1) mm^3^ and matrix size of (240 × 240 × 155) was preserved across all scans.

In accordance with IBSI guidelines [37], image preprocessing was conducted to support reproducibility, including Z-score normalization and uniform voxel resampling. The following two-phase approach was employed for tumor segmentation in the BraTS 2020 and RHUH-GBM datasets: automatic segmentation using DL techniques, followed by confirmation from neuroradiologists to ensure compliance with clinical standards [29,32]. For each patient, 1980 RFs were extracted, derived from a combination of four MRI modalities and three tumor regions, with 165 features computed per region. The extraction process was conducted using the MATLAB-based version of the Spaarc Pipeline for Automated Analysis and Radiomics Computing (SPAARC V1.0, https://www.spaarc-radiomics.io (accessed on 1 May 2026)) [38,39]. Tumor characteristics such as shape, texture, and intensity were quantified through radiomic feature extraction. To promote reproducibility and consistency across datasets, all features were derived using a three-dimensional (3D) methodology and standardized in compliance with IBSI guidelines. Details of the preprocessing parameters and extracted RFs are presented in Table S1 of the Supplementary Materials.

### 2.4. Identification of Clinical and Radiomic Signatures

**Figure 2:**
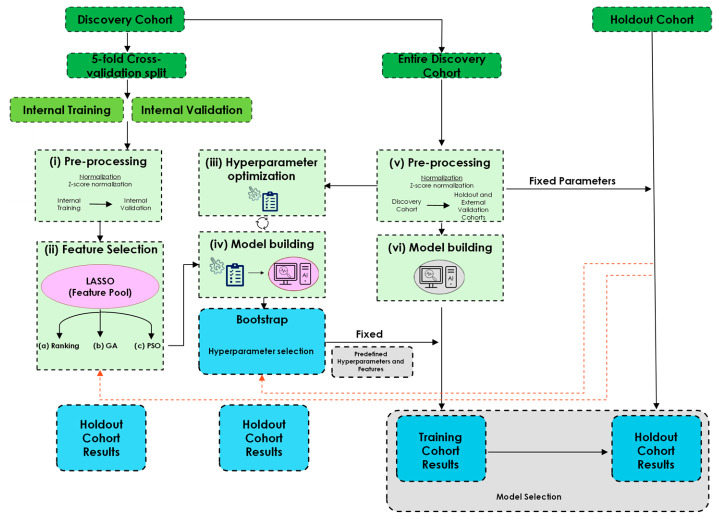
The radiomic workflow for feature selection methods and hyper-parameter optimization.

To promote methodological rigor and reduce overfitting, model development followed a structured pipeline that employed three complementary feature selection approaches. The modeling framework, as depicted in Figure 2, consisted of the following six stages: (i) feature preprocessing, (ii) feature selection, (iii) hyperparameter tuning, (iv) model training with internal validation on the discovery cohort and the holdout cohort, (v) feature preprocessing for the final model and (vi) model building with fixed parameters. All steps were conducted using five-fold cross-validation except for stages (v) and (vi). By using the selected model and feature set, the hyperparameters were optimized with nested cross-validation via “gridsearchcv” and the bootstrapping method on the entire discovery cohort. Additionally, the results were validated on the holdout cohort to select feature sets for each feature selection method. Detailed illustrations of each step are provided in the Supplementary Materials: LASSO-RANK (Figure S1), LASSO-GA (Figures S2), and LASSO-PSO (Figure S3 and descriptive text), while the following sections describe in detail each stage of the modeling framework, outlining the processes of feature preprocessing, feature selection, hyperparameter tuning, and model training with internal validation.

(i) To address feature scale variability, Z-score normalization was conducted on the discovery dataset. The resulting parameters were then used to normalize the holdout test and external validation datasets.
(ii) The feature selection methods cover the following three methods: one traditional approach and two novel variations that employ algorithms based on utilizing nature-inspired feature selection algorithms [40]. The established LASSO-RANK algorithm [41], outlined in Leger et al. [42], provided the fundamentals, while this study proposed a novel two-phase hybrid feature selection method. The first step of the new method was a key modification of LASSO-RANK, which excluded only the frequency-based feature ranking step after generating a feature pool. This pool was subsequently utilized using nature-inspired algorithms, specifically GA [43] and PSO [44], to enhance the feature selection step. The proposed models are named LASSO-GA and LASSO-PSO. The LASSO-RANK method, used as the baseline approach, employed a five-fold cross-validation strategy to identify a group of up to nine RFs, ranging from 2-feature to 9-feature subsets. In the novel two-phase approach, either GA or PSO was used to refine the feature pool obtained from LASSO regression. In contrast to LASSO-RANK’s deterministic feature selection, GA and PSO apply stochastic (random), iterative search strategies to optimize feature subset selection. The details of hyperparameter configurations for these algorithms are provided in Table S2. The selected estimator was LASSO with a negative mean squared error metric for GA and PSO algorithms. Risk stratification was conducted utilizing the following two survival analysis models: regularized Cox regression (Cox-LASSO) [41] and Random Survival Forests (RSFs) [45]. These frequently used models [46] have been tailored for survival analysis, optimizing to effectively handle censored time-to-event data.
(iii) Model hyperparameters were tuned via bootstrap resampling on the discovery cohort to minimize overfitting and improve the model’s risk-stratification performance on unseen data.
(iv) In line with van Timmeren et al. [12], the total feature number, including patient age as a clinical variable, was limited to 10. Thus, RFs can be as low as 2 features [11,26] and up to 9 features in total, except for a single clinical variable. LASSO-RANK identified 2-feature to 9-feature subsets per fold through 5-fold cross-validation. The RFs of 8 variant feature pools, ranging from 2-feature to 9-feature pools, were then ranked within their individual feature pools based on selection frequency. To assess risk stratification performance, model validation was performed using 200 bootstrap resampling iterations on the discovery cohort with the selected feature subsets. Additionally, LASSO-GA and LASSO-PSO also utilized the same 8 variant feature pools without ranking steps to select the final feature subsets, applying the same strategy for model validation.

### 2.5. Statistical Analysis

The C-index was used as the primary performance metric to evaluate robustness and predictive accuracy, with model development and optimization conducted on the discovery cohort. Performance evaluation was carried out on the following two datasets: the holdout portion of BraTS 2020 and an external validation set (RHUH-GBM). By implementing a two-stage validation, the study aimed to assess both model performance and generalizability.

Survival distributions were compared using the log-rank test in the discovery and both the holdout test set and the external validation cohort. To assess statistical differences in continuous variables, the Mann–Whitney U test was applied between the discovery cohort and both the holdout test set and the external validation cohort. The Kaplan–Meier curve was used to assess risk stratification, with patients divided into low- and high-risk groups according to the median risk score as the cut-off. The selected cut-off value from the discovery cohort was applied to unseen cohorts. To determine the statistical difference in survival outcomes between high- and low-risk groups, the log-rank test was employed. The prognostic models’ stratification performance was measured using the C-index, with 200 bootstrap iterations performed across the discovery, holdout test, and external validation cohorts to estimate the 95% confidence interval (CI) [47].

All statistical and survival analyses were performed in Python v3.10, with *p* < 0.05 considered statistically significant. Image preprocessing and analysis steps are outlined in Figure 2. Permutation feature importance was used for feature relevance assessment via Scikit-learn v1.5.2. PSO and GA were implemented using ps-optimize v2.0.4 and sklearn-genetic v0.6.0, respectively.

## 3. Results

The clinical characteristics of the discovery, holdout test, and external validation cohorts were summarized in Table S3 of the Supplementary Material. The discovery cohort presented a median OS of 12.05 months, while the holdout test cohort exhibited a median OS of 14.44 months. Statistical analysis indicated that the difference in median OS between these cohorts was not significant (*p* = 0.58). The external validation cohort had a median OS of 12.13 months, which was not significantly different from that of the discovery cohort (*p* = 0.59). The LASSO-RANK and LASSO-GA methods achieved peak performance with a five-feature pool and a seven-feature pool, respectively. In comparison, LASSO-PSO benefited from larger feature sets, requiring a nine-feature pool to reach its optimal performance level. To address multicollinearity, an initial step was carried out before the feature selection process. To reduce feature redundancy, Spearman correlation coefficients were calculated across all RFs. Subsequently, features demonstrating high correlation (ρ > 0.95) were excluded, yielding a refined feature set of 767 RFs.

RFs selected across all five cross-validation folds were aggregated to provide the final feature pools. Duplicate features were then removed. This process yielded 15 RFs for LASSO-RANK (from a five-feature pool), 16 RFs for LASSO-GA (from a seven-feature pool), and 18 RFs for LASSO-PSO (from a nine-feature pool). Each feature subset was assessed with RFs-only models on the holdout cohort to prevent data leakage and reduce bias (Supplementary Materials Table S5). For the LASSO-RANK method, the final model utilized five features demonstrating the highest selection frequency, as the best performance was previously achieved with a configuration of the five-feature pool. Internal validation guided the final selection of feature sets for LASSO-GA and LASSO-PSO, which included four RFs and 10 RFs respectively, as shown in Table 1.

Cox-LASSO and RSF hyperparameters were tuned using 200 bootstrap iterations on the entire discovery cohort, based on the selected RFs and patient age as the clinical feature. For every combination of model and feature selection method, the final hyperparameter configurations were optimized using k-fold cross-validation and bootstrap techniques; all hyperparameter configurations are presented in Table S4 of the Supplementary Materials. The development of prognostic models included the full discovery cohort, combining patient age info with the selected RFs to create an integrated clinical-radiomic signature. Through the application of LASSO-based feature selection techniques, a subset of robust RFs was identified across diverse MRI sequences, preserving performance with fewer features.

**Figure 3:**
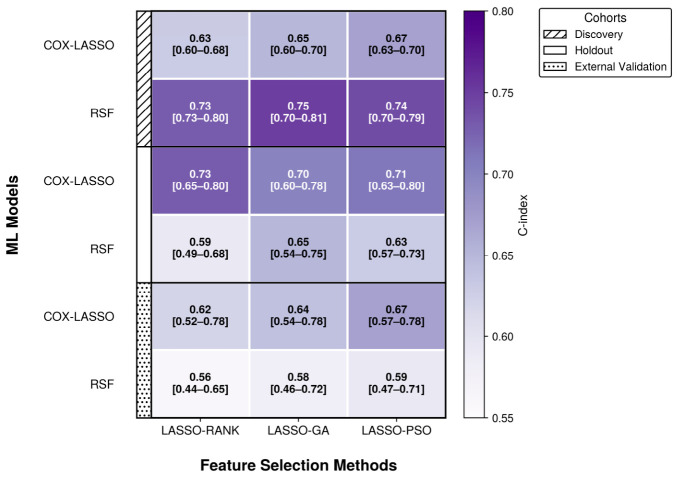
The C-index values for each model on each feature selection method and their corresponding ML algorithms were assessed for GBM time-to-event analysis. The results of clinical-radiomic models are presented as follows: model performance was evaluated across the following three cohorts: discovery, holdout, and external validation. Higher values are highlighted with darker colors.

RSF models performed the best on the discovery cohort, with C-index values of 0.73,0.75 and 0.74. However, their performance dropped significantly on the external validation cohort, with the C-index values of 0.56, 0.58, and 0.59. Therefore, RSF results were excluded from further evaluation (Figure 3). Recognized for its high interpretability [48], the Cox-LASSO model achieved the highest C-index values of 0.67 on the external validation cohort when using features selected by only LASSO-PSO (Figure 3). Additionally, the models achieved high C-index values on internal validation (the discovery cohort and the holdout cohort) when using features selected by LASSO-PSO or LASSO-GA (Figure 3). According to the evaluation on the external validation dataset (Figure 3), the prognostic model achieved a C-index of 0.67 using LASSO-PSO selected features. The result of the model is higher than that of the model with LASSO-GA (C-index = 0.64), representing robustness with LASSO-PSO selection. Despite demonstrating the best discovery cohort performance with a C-index of 0.75, the best-performing RSF model using LASSO-GA generalized poorly to the external validation dataset. Additionally, the result of the Cox-LASSO model, using only the age feature, did not generalize well on the external validation cohort (Supplementary Materials Table S5).

The Cox-LASSO model that was built using 10 RFs (cf. Table 2) exhibited notable predictive accuracy within the discovery cohort, as reflected by a C-index of 0.64 with a 95% CI of 0.58–0.70. The texture feature dzm_zdnu_3D, using TC label, had the highest hazard ratio (HR = 1.15) in the 10-feature radiomic model (cf. Table 2), with a 95% CI of 0.91–1.45. This feature quantifies the distribution uniformity of zone frequencies through spatial distances. Low scores indicate zone homogeneity in the tumor; high scores indicate clustering and greater intertumoral heterogeneity. The selected feature set comprised an equal distribution between MRI sequence-dependent and shape-based features as follows: 50% (5/10) were morphological (shape-based) features not tied to a specific MRI sequence, and the remaining 50% were extracted from FLAIR (4/10) and T1 (1/10) sequences. These RFs exhibited low inter-correlation (Spearman’s ρ < 0.3), suggesting independent contributions. On the holdout test dataset, the radiomic model achieved a C-index of 0.62 (95% CI: 0.53–0.73), demonstrating moderate prognostic performance in Table 2. morph_comp_1, a shape-based feature, emerged as the most notable predictor, exhibiting the highest hazard ratio (HR) of 2.83 (95% CI: 0.92–8.68), as documented in Table 2. By evaluating the geometrical conformity of the targeted ROI to a perfect spherical morphology, this feature serves as a metric for characterizing tumor morphological compactness.

The clinical-radiomic model, combining age and 10 RFs, achieved the best C-index in the discovery dataset (0.67, 95% CI: 0.62–0.72). The model yielded a C-index of 0.71 (95% CI: 0.63–0.80) on the holdout test dataset, indicating good prognostic performance (Table 3). Notably, two specific RFs emerged as strong predictors of high-risk status, each exhibiting a hazard ratio (HR) greater than 2.0. Szm_lgze_3D, obtained from FLAIR sequence data and corresponding to ET label, revealing an HR of 2.57 (95% CI: [0.11–57.27]). When limiting feature numbers, this feature will be excluded in the final feature set due to the substantially wide confidence interval. Simultaneously, morph_pca_flatness, extracted from the TC label, demonstrated an HR of 2.21 (95% CI: [1.21–4.03]). The texture feature szm_lgze_3D captures the frequency and extent of zones with low gray-level intensities within the ET region, providing insight into lesion heterogeneity. Then, morph_pca_flatness, a shape-based feature, represents the ratio of the least principal axis length and the major principal axis length. The metric’s value exhibits convergence toward one as the TC label geometrically approximates a perfect spherical configuration, thus representing enhanced shape uniformity. In the external validation dataset, the clinical-radiomic model achieved a C-index of 0.67 (95% CI: [0.57–0.78]).

**Figure 4:**
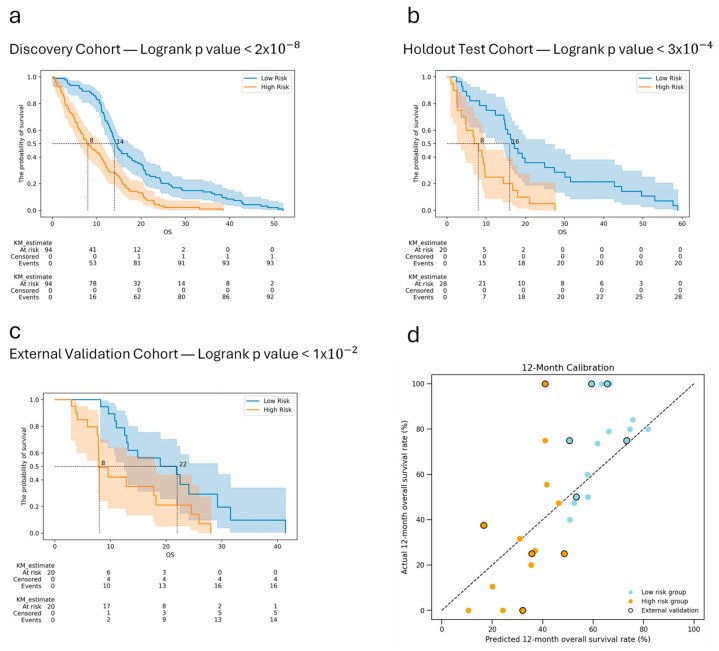
Kaplan–Meier curves display survival differences in the (**a**) training (discovery), (**b**) holdout test and (**c**) external validation datasets, categorized into low- and high-risk groups by the Cox–LASSO model. The small *p*-values suggest strong statistical reliability in distinguishing between risk groups. The dashed lines in (**a**–**c**) indicate the time point at which an individual has a 50% chance of survival for each risk group within the cohort. (**d**) Calibration curve assessing the agreement between predicted and observed survival probabilities at 12 months.

The log-rank test assessed survival differences between low- and high-risk groups based on a Kaplan–Meier cut-off of 0.0096 (cf. Supplementary Materials, Table S6). The model demonstrated significant stratification ability, revealing distinct survival outcomes between predicted risk groups in the discovery, holdout test, and external validation datasets (*p* = 2 × 10^−8^, *p* = 3 × 10^−4^, and *p* = 0.01, respectively; cf. Figure 4a–c). The Kaplan–Meier survival curves demonstrated the model’s consistent ability to differentiate between high- and low-risk patient groups across all datasets. Statistical analysis confirmed significant differences between risk groups, which were also evident in the external validation cohort. Also, calibration curve analysis was added at the 12-month survival prediction (cf. Figure 4d).

The permutation importance for each feature is shown in Figure S4 of the Supplementary Materials with further information such as feature weights and the Kaplan–Meier threshold (reported in Supplementary Materials, Table S6). According to feature importance analysis based on the final model’s weights, the clinical feature, age, emerged as the most influential predictor overall. Among the RFs specifically, morph_pca_maj_axis was identified as the most significant contributor to the model’s output. This morphological feature evaluates the maximum axial dimension of the ROI-defining ellipsoid for the TC label, determined via principal component analysis and represented by the major eigenvalue (λ_major_). Analytical results indicated that both patient age and the morph_pca_maj_axis feature significantly elevated the likelihood of assignment to the high-risk patient group. The collective effect of these factors indicates that older age and an increased major axis length of the ellipsoid encompassing the ROI may serve as significant predictors of poor prognosis and increased tumor aggressiveness.

**Figure 5:**
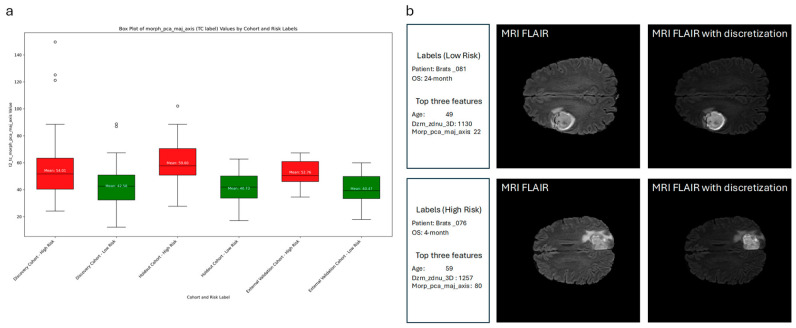
Feature-level model interpretation using the most significant radiomic feature, morph_pca_maj_axis. (**a**) Box plots illustrate the stratification of risk groups across the discovery, holdout test, and external validation cohorts. (**b**) Patient-level analysis of the discovery cohort. Data representation includes overall survival (OS) alongside the top three features from the final clinical-radiomic model. RFs were extracted from MRI FLAIR scans, shown with and without discretization.

To enhance model interpretability, we defined dual thresholds of the top three features based on the final model. From Figure 5a, the thresholds of morph_pca_maj_axis, the most significant radiomic feature, were established using the final model on the discovery cohort, defining the low-risk group as feature values < 43 (corresponding to the Q2–Q3 quartiles) and the high-risk group as values > 54 (corresponding to the Q1–Q2 quartiles). Additionally, specific thresholds were determined for the following remaining top three features: age (low-risk < 57; high-risk > 67) and dzm_zdnu_3D (low-risk < 873; high-risk > 1504). Detailed distributions relative to these thresholds and additional examples are available in the Supplementary Materials (Supplementary Figures S5 and S6, Table S7). From Figure 5b, the results of the discovery cohort revealed a relationship between these features. The patient (top) was stratified into the low-risk group. This patient is characterized by a high dzm_zdnu_3D value (1130 > 873) and low values for both morph_pca_maj_axis (22 < 43) and age (49 < 57) relative to the low-risk thresholds. Despite the presence of relatively high tumor heterogeneity (dzm_zdnu_3D), patients may exhibit long survival provided that the tumor’s major axis length (morph_pca_maj_axis) remains low and the patient is of a younger age. The patient in Figure 5b, who was stratified into the high-risk group, was characterized by a high morph_pca_maj_axis value (80 > 54), notwithstanding low age (59 < 67) and low dzm_zdnu_3D (1257 < 1504) values, relative to the high-risk thresholds. The negative prognostic impact of the tumor’s major axis length (morph_pca_maj_axis) on survival was significant, outweighing younger patient age or low tumor heterogeneity (dzm_zdnu_3D). However, the model does not directly show biological causality or explain the underlying tumor mechanisms, highlighting the need for further research on the related biological pathways. Additionally, time-dependent AUC and proportional hazard assumptions were performed to further assess model performance (Supplementary Materials Figures S7 and S8).

## 4. Discussion

In this research, we developed a risk stratification model for GBM based on preoperative multimodal MRI scans that included both a clinical factor and RFs. Ten RFs were selected using the LASSO-PSO selection method, which was derived from FLAIR and T1 MRI sequences. The analysis showed that morphological features formed most of the selected feature set (5/10 RFs), while texture-based features extracted from FLAIR sequences constituted the second-largest component (4/10 RFs). T1 provided just one first-order feature. All RFs, except for two texture features derived from ET and WT labels, were obtained from the TC label. In the holdout test dataset, the clinical-radiomic model achieved a C-index of 0.71, demonstrating effective and statistically significant differentiation between low- and high-risk groups. The external validation confirmed the model’s robustness, with a C-index of 0.67 and a significant log-rank test result confirming its ability to generalize.

We compared the findings of this study with those from earlier works. Only studies using RFs (engineered or deep features) and clinical data were included. Excluded from the analysis were studies that utilized subjective measures (e.g., VASARI) or RFs lacking reproducibility standards prior to the initial IBSI study [37]. The comparison was further restricted to studies addressing GBM and applying time-to-event survival analysis for OS prediction. A detailed evaluation of the methodological approaches determined important limitations, particularly in single-center studies, potentially decreasing their applicability across heterogeneous clinical settings. The study by Fathi et al. [15] included a single-center dataset, while the study by Gomaa et al. [13] enhanced their study’s impact through external validation. We addressed study limitations by expanding the study cohort, incorporating the following two datasets sourced from different centers: BraTS 2020 and RHUH-GBM. While this approach improves the study’s reliability, residual biases may still be present, highlighting the need for further validation in diverse clinical settings.

The work by Verduin et al. [14], the assessment of prognostic and predictive value of MRI image analysis in GBM yielded a C-index of 0.69 using five radiomic and five clinical features. In contrast, Duman et al. [26] achieved the same performance with only three features (two RFs and a single clinical feature). Notably, both studies lacked open-access external validation, while this work included open-access external validation. The study conducted by Fathi et al. [15] achieved a C-index of 0.70, employing a model consisting of 24 RFs and three clinical variables, which might decrease the interpretability of the model. Additionally, Gomaa et al. [13] reported C-indexes of 0.71, 0.67, and 0.62 across the UPenn, UCSF, and RHUH-GBM cohorts, respectively. However, the study, utilizing numerous deep features and four clinical variables, dramatically impacts the model interpretability. Similarly, Verduin et al. [14] reported a C-index of 0.69 using convolutional filters, which were not reported to be IBSI compliant [49]. On the other hand, our study aimed to limit the feature number while utilizing IBSI guidelines for radiomic feature extraction. The radiomic model developed by Al-Tashi et al. [17], which combined SI algorithms, DL-based survival analysis, and 49 RFs, demonstrated modest predictive capacity (C-index: 0.61) and failed to achieve statistical significance (*p* = 0.14). In contrast to the common use of Cox regression in radiomics studies [46], Al-Tashi et al. and Gomaa et al. examined SwarmDeepSurv and transformer-based DL architectures for survival analysis. Although these methods demonstrate potential, their lack of transparency presents considerable challenges for clinical integration. We implemented an SI-based algorithm to collect a limited set of features, thereby enhancing the interpretability of ML models while maintaining strong predictive and stratification performance.

Using only 10 RFs from FLAIR and T1 sequences, together with the clinical variable age, our study achieved C-indices of 0.71 (the holdout test cohort) and 0.67 (the external validation cohort), matching or outperforming prior studies while relying on a highly compact feature set. This study found that our method, which utilizes an interpretable Cox-LASSO model and reproducible RFs compliant with IBSI standards [37], achieved superior performance metrics when evaluated on identical external validation data (RHUH-GBM) compared to the DL-based study by Gomaa et al. [13]. Our novel SI-enhanced method, LASSO-PSO, identified 10 RFs, including one clinical feature. The morphological feature morph_pca_maj_axis demonstrated the most significant impact within the validation set. The model demonstrated strong cross-institutional utility through optimized feature selection, maintaining predictive accuracy across varied clinical settings and multiple sourced datasets. The assessment of feature weights and importance (cf. Supplementary Table S6) suggests that high-risk patients exhibit an increased axis length (the major eigenvector extracted from principal component analysis), defined by morph_pca_maj_axis, a morphological feature. Derived from the Gray-Level Distance Zone Matrix (GLDZM), dzm_zdnu_3D feature, Zone Distance Non-Uniformity (ZDNU), computes the degree of uniformity in zone distribution at varying distances, providing insight into the spatial heterogeneity of the tumor. Higher values of the feature indicated greater tumor heterogeneity, which was associated with an increased likelihood of aggressive tumor types and assignment to the high-risk patient category. The TC label was utilized to quantify the two most significant RFs. Within this feature subset, dzm_zdnu_3D was extracted from FLAIR sequences, highlighting its important role in the characterization of tumor aggressiveness. In this study, LASSO-PSO outperformed LASSO-RANK and LASSO-GA in selecting an optimal feature subset for radiomic modeling. This result aligns with the comprehensive review of Rostami et al. [40] of SI-based feature selection methods, which shows the PSO algorithm’s ability to select a minimum number of features without compromising model performance compared to other algorithms.

To the best of our knowledge, this is among the first studies to apply an SI-based feature selection with traditional ML models that achieves statistically significant risk stratification in GBM time-to-event analysis. Despite utilizing a limited set of clinical and RFs, the proposed model achieved performance levels that were superior to previous studies. Following the guidelines set by van Timmeren et al. [12], we excluded RFs with low feature importance (<0.01), as shown in Supplementary Material Figure S4, from the initial set of 10 RFs. The excluded features were szm_lgze_3D, derived from FLAIR sequence data using the TC label, as well as morph_vol_dens_aee and morph_area_dens_aee, both of which were extracted using the TC label. Also, szm_lgze_3D, derived from FLAIR sequence data using the ET label, was excluded due to the substantially wide confidence interval (Table 3). This methodological approach resulted in a finalized feature set of seven features, which included age as a clinical variable. This revised model preserved discriminative power, with C-indices of 0.70 (BraTS 2020) and 0.68 (RHUH-GBM). The constraint, limiting the analysis to feature subsets of 3–10 features, significantly impacted the features evaluated. However, the potential of the SI-based selection method represents a promising avenue to enhance performance when applying it to an expanded set of RFs in the future. Notably, excluding the non-robust szm_lgze_3D feature from the ET label improved model performance across the RHUH-GBM dataset. The instability of this feature likely reflects segmentation variability. Another limitation is heterogeneity in MRI acquisition protocols or scanners. These limitations can be addressed in future multi-institutional studies via automated segmentation and the ComBat harmonization method.

Potential retrospective sampling bias was systematically mitigated through the utilization of data derived from multiple institutions (the BraTS 2020 dataset), with further evaluation provided with external validation procedures (RHUH-GBM dataset). However, the retrospective design and absence of key clinical variables (e.g., KPS, genetic markers, and extent of resection) present notable limitations. In the future, model optimization could cover integrating expanded clinical parameters (extent of resection, genetic markers, MGMT, KPS, etc.), additional imaging modalities (PET, CT, and ultrasound), and other omics (genomics, pathomics). Due to having multiple features from different sources, feature fusion methods could present a promising research direction to improve model generalizability [50]. Following the IBSI standardization of convolutional filters [49], future studies could focus on implementing these standardized filters. Additionally, we aim to investigate the utility of nature-inspired approaches for ML- or DL-based model development with hyperparameter optimization, specifically exploring their potential to improve predictive performance.

## 5. Conclusions

This study presents an interpretable clinical-radiomic model for predicting OS in GBM, developed using a novel SI-based hybrid feature selection method and validated across multi-institutional datasets. By integrating this feature selection strategy with a Cox-LASSO framework, the model may support future clinical translation following further prospective validation. Future work incorporating additional data sources and larger, diverse cohorts may further enhance predictive performance and support the adoption of radiomics in clinical decision-making for GBM.

## Figures and Tables

**Table 1:** The selected RFs for each feature selection method, with their respective MRI sequences and labels indicated in parentheses. Notably, morphological features are associated with contour morphology (shape) rather than a specific MRI sequence. The MRI sequences used for radiomic feature extraction are specified in parentheses (e.g., FLAIR sequence, T1 sequence), along with the regions of interest (ROIs) utilized for feature extraction (e.g., ET label, TC label, WT label).

LASSO-RANK (5 Features)	LASSO-GA (4 Features)	LASSO-PSO (10 Features)
morph_volume (TC label) morph_av (TC label) morph_pca_flatness (TC label) morph_pca_maj_axis (TC label) morph_pca_elongation (TC label)	morph_area_dens_aabb (TC label) ngl_dc_entr_3D (FLAIR sequence, WT label) dzm_zdnu_3D (FLAIR sequence, TC label) stat_skew (T1 sequence, TC label)	morph_pca_maj_axis (TC label) morph_pca_flatness (TC label) morph_comp_1 (TC label) morph_vol_dens_aee (TC label) morph_area_dens_aee (TC label) ngl_dc_entr_3D (FLAIR sequence, WT label) dzm_zdnu_3D (FLAIR sequence, TC label) szm_lgze_3D (FLAIR sequence, ET label) szm_lgze_3D (FLAIR sequence, TC label) stat_skew (T1 sequence, TC label)

**Table 2:** Univariate (clinical model) and multivariate (radiomic model) Cox regression analysis for discovery and holdout test datasets.

		Discovery Cohort	Holdout Test Cohort	Discovery Cohort		Holdout Test Cohort	
Model	Variable	HR [95% CI]	HR [95% CI]	*p*-Value	C-Index	*p*-Value	C-Index
Clinical Model	Age	1.32 [1.12–1.55]	1.78 [1.35–2.35]	3 × 10^−2^	0.59 [0.54–0.66]	1 × 10^−4^	0.71 [0.63–0.80]
Radiomic Model	morph_pca_maj_axis (TC label)	1.08 [0.80–1.45]	2.05 [0.89–4.72]	6 × 10^−4^	0.64 [0.58–0.70]	2 × 10^−2^	0.62 [0.53–0.71]
	morph_pca_flatness (TC label)	1.13 [0.87–1.48]	1.38 [0.79–2.41]				
	morph_comp_1 (TC label)	0.92 [0.63–1.35]	2.83 [0.92–8.68]				
	morph_vol_dens_aee (TC label)	0.88 [0.60–1.31]	0.41 [0.13–1.28]				
	morph_area_dens_aee (TC label)	1.13 [0.85–1.50]	2.39 [0.84–6.76]				
	ngl_dc_entr_3D (FLAIR sequence, WT label)	0.91 [0.76–1.09]	0.40 [0.22–0.74]				
	dzm_zdnu_3D (FLAIR sequence, TC label)	1.15 [0.91–1.45]	1.14 [0.66–1.98]				
	szm_lgze_3D (FLAIR sequence, ET label)	0.88 [0.68–1.15]	0.49 [0.03–8.86]				
	szm_lgze_3D (FLAIR sequence, TC label)	0.93 [0.75–1.14]	1.25 [0.23–6.82]				
	stat_skew (T1 sequence, TC label)	0.86 [0.74–0.98]	1.06 [0.75–1.51]				

**Table 3:** Multivariate Cox regression analysis for clinical-radiomic model.

		Discovery Cohort	Holdout Test Cohort	Discovery Cohort		Holdout Test Cohort	
Model	Variable	HR [95% CI]	HR [95% CI]	*p*-Value	C-Index	*p*-Value	C-Index
Clinical-Radiomic Model	Age	1.36 [1.14–1.63]	1.92 [1.31–2.82]	2 × 10^−8^	0.67 [0.62–0.72]	3 × 10^−4^	0.71 [0.63–0.80]
	morph_pca_maj_axis (TC label)	1.23 [0.91–1.66]	1.72 [0.72–4.13]				
	morph_pca_flatness (TC label)	1.17 [0.90–1.54]	2.21 [1.21–4.03]				
	morph_comp_1 (TC label)	0.90 [0.61–1.33]	1.60 [0.54–4.77]				
	morph_vol_dens_aee (TC label)	0.94 [0.63–1.39]	0.36 [0.12–1.09]				
	morph_area_dens_aee (TC label)	1.04 [0.78–1.40]	1.98 [0.73–5.40]				
	ngl_dc_entr_3D (FLAIR sequence, WT label)	0.87 [0.73–1.04]	0.54 [0.27–1.05]				
	dzm_zdnu_3D (FLAIR sequence, TC label)	1.14 [0.91–1.44]	1.06 [0.61–1.85]				
	szm_lgze_3D (FLAIR sequence, ET label)	0.85 [0.65–1.12]	2.57 [0.11–57.27]				
	szm_lgze_3D (FLAIR sequence, TC label)	0.99 [0.81–1.22]	0.53 [0.09–3.17]				
	stat_skew (T1 sequence, TC label)	0.88 [0.76–1.00]	0.92 [0.65–1.29]				

## Data Availability

The source code of the LASSO-PSO is available at GitHub public repository (https://github.com/krmdmn/si-feature-selection-for-radiomics (accessed on 1 June 2026)). The BraTS data used in this study are openly available and can be accessed from the following source (https://www.kaggle.com/datasets/awsaf49/brats20-dataset-training-validation (accessed on 1 June 2026)). RHUH-GBM data used in this study are also openly available and can be accessed from The Cancer Imaging Archive (TCIA) website (https://www.cancerimagingarchive.net/, accessed on 1 June 2026).

## Supplementary Materials

The following supporting information can be downloaded at https://www.mdpi.com/article/10.3390/cancers18121888/s1, Table S1: IBSI standardized preprocessing parameters for radiomic analysis; Table S2: Hyperparameters for PSO and GA were set based on the example source codes, with the exception of “Maximum Features”, which was adjusted to the total feature number for each feature pool for GA, leading to improved model performance; Table S3: Characteristics of clinical variables for discovery and holdout test and external validation datasets; Table S4: Hyperparameters for each model and feature selection method from 200 bootstrapped iterations of the training (discovery) dataset; Table S5: RFs-only Models and n-feature selection based on the C-index values for holdout dataset; Table S6: Feature importance and weights of each feature in the final clinical-radiomic mode; Table S7: Dual thresholds of top three features from the final model; Figure S1: LASSO-RANK feature selection framework: Univariate Analysis included correlation analysis and Lasso Cox. The last step was conducted by ranking of features based on their selection frequency; Figure S2: (a) LASSO-GA feature selection framework: Univariate Analysis included correlation analysis and Lasso Cox. The last step was conducted by the selection of features based on GA. (b) Steps of the GA-based feature selection workflow with the feature pool from LASSO; Figure S3: (a) LASSO-PSO feature selection framework: Univariate Analysis included Correlation analysis and Lasso Cox. The last step was conducted by the selection of features based on PSO. (b) Steps of PSO-based feature selection workflow with the feature pool from LASSO; Figure S4: Feature importance for the final clinical-radiomic model; Figure S5: Feature-level model interpretation using Dzm_zdnu_3D (FLAIR sequence, TC label); Figure S6: Feature-level model interpretation using Age; Figure S7: Time-dependent AUC for train (discovery), holdout, external cohorts; Figure S8: Proportional hazard assumptions (Scaled Schoenfeld).
