# Supplementary material for: A Novel Swarm Intelligence-Driven Feature Selection for Interpretable Machine Learning in Multiparametric MRI-Based GBM Overall Survival Analysis
Source: Cancers (Basel). 2026 Jun 10;18(12):1888. doi: 10.3390/cancers18121888 (PMC13296785; doi:10.3390/cancers18121888)
Supplement: Supplementary file 1 [file cancers-18-01888-s001.zip › cancers-4309738-supplementary.pdf]

Supplementary Materials: A Novel Swarm Intelligence-Driven Feature Selection for Interpretable Machine Learning in Multiparametric MRI-Based GBM Overall Survival Analysis

Parameters	Values
Voxel Spacing	[1, 1, 1]
Interpolation Method	Spline
Bin method	Fix Bin Number (FBN)
Bin value	64
Analysis Type	3D
Feature Families	Morphology (MORPH), Statistical (STATS), Intensity Histogram (IH), Intensity-volume histogram (IVH), Grey Level Co-occurrence Matrix (GLCM), Grey Level Run Length Matrix (GLRLM), Grey Level Size Zone Matrix (GLSZM), Grey Level Distance Zone Matrix (GLDZM), Neighbourhood Grey Level Dependence Matrix (NGLDM), Neighbourhood Grey Tone Difference Matrix (NGTDM),
**Texture Matrix**	**Parameters**
Grey Level Co-occurrence Matrix (GLCM)	Distance = 1 voxel, Directions = 13 unique directions of the 26-connected neighbours, Matrix merging method = Merged GLCMs for each direction and calculated features on single matrix.
Grey Level Run Length Matrix (GLRLM)	Distance = 1 voxel, Directions = 13 unique direction vectors, Matrix merging method = Merged GLRLMs for each direction and calculated features on single matrix.
Grey Level Size Zone Matrix (GLSZM)	Connectivity for all 26 neighbours in 3D.
Grey Level Distance Zone Matrix (GLDZM)	Connectivity for all 26 neighbours in 3D.
Neighbourhood Grey Tone Difference Matrix (NGTDM)	Distance = 1 voxel.
Neighbourhood Grey Level Dependence Matrix (NGLDM)	Distance = 1 voxel.

Table S-1 IBSI standardized preprocessing parameters for radiomic analysis.

Parameter	Value	Parameter	Value
Algorithm	PSO	Algorithm	GA
Particle Number	30	Population Number	50
Estimator	LASSO	Estimator	LASSO
CV	3	CV	5
Scoring	Negative Mean Squared Error	Scoring	Negative Mean Squared Error
Max Iteration	10	Crossing-over Probability	0.5
		Mutation Probability	0.2
		Number of Generations	40
		Maximum Features	Total feature number from a feature pool
		Population Number	50

Table S-2 Hyperparameters for PSO and GA were set based on the example source codes, with the exception of 'Maximum Features', which was adjusted to the total feature number for each feature pool for GA, leading to improved model performance.

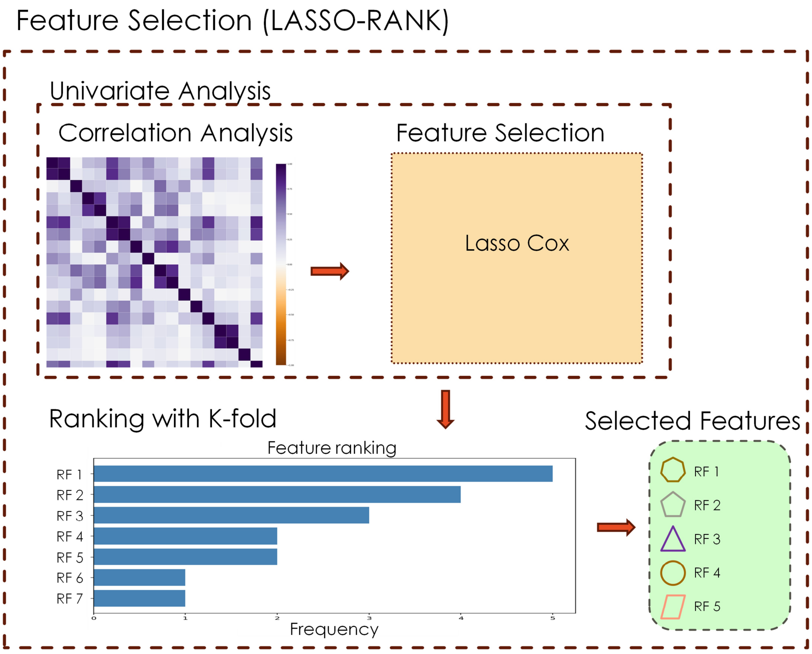

Figure S-1 LASSO-RANK feature selection framework: Univariate Analysis included Correlation analysis and Lasso Cox. The last step was conducted by ranking of features based on their selection frequency.

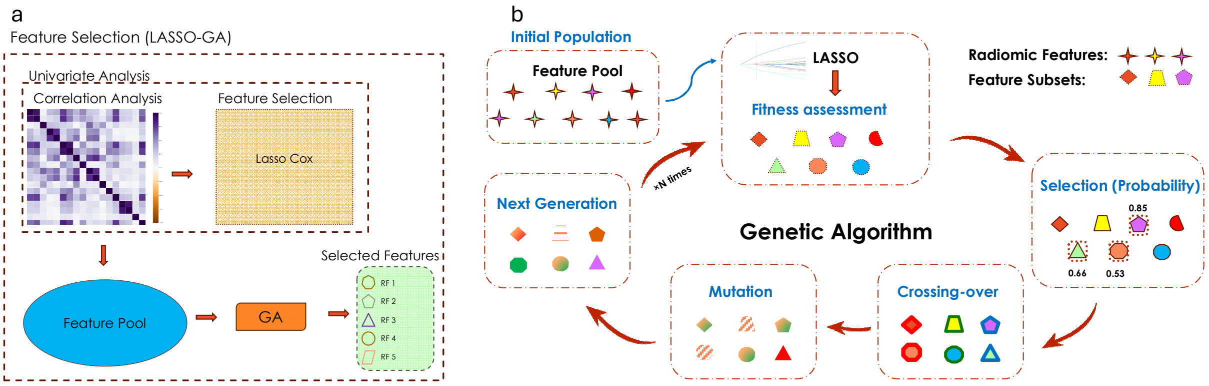

Figure S-2 a) LASSO-GA feature selection framework: Univariate Analysis included Correlation analysis and Lasso Cox. The last step was conducted by the selection of features based on GA. b) Steps of the GA-based Feature Selection Workflow with the feature pool from LASSO. The process starts by sampling the feature pool to create initial subsets. Next, it uses fitness scores to pick the best feature subsets and swap features out to improve the results in the next iteration.

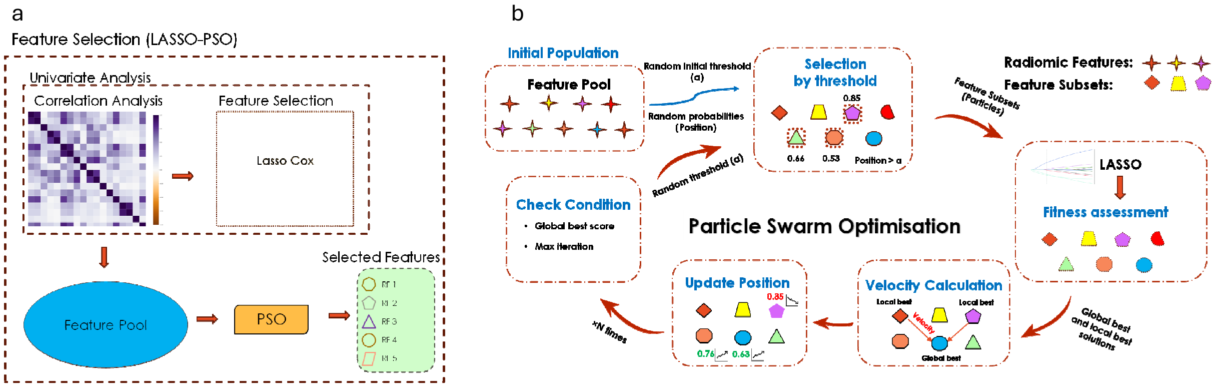

FigureS-3 a) LASSO-PSO feature selection framework: Univariate Analysis included Correlation analysis and Lasso Cox. The last step was conducted by the selection of features based on PSO. b) Steps of PSO-based feature selection workflow with the feature pool from LASSO. It creates a random initial threshold and random probabilities (positions) for each feature subset and starts by sampling the feature pool to create initial subsets. Next, it uses fitness scores to update the positions of the feature subsets and swap features out to improve the results in the next iteration.

Simple PSO Algorithm Specification for Feature Selection

The proposed implementation constitutes a simplified variant of standard Particle Swarm Optimization (PSO). The proposed algorithm simplifies these complexities, relying instead on a direct, mathematically calculated trajectory toward the final feature subset.

The complete lifecycle of this custom algorithm is detailed below.

1. Swarm Initialization

- Probability Matrix Formulation: The algorithm initializes a probability matrix representing the entire swarm. For a swarm of N particles (from the initial feature pool) and a dataset of F features, an N × F matrix is generated. Each element (feature) is initialized with a random probability between 0 and 1, representing the initial "position" of the particles.

- Velocity Matrix Initialization: An initial velocity matrix of identical dimensions is generated and populated entirely with zeros.

- Global Tracker Initialization: The tracking variables for the highest evaluation metric (global_best) and its corresponding feature subset (global_best_particle) are initialized as null values.

2. Initial Position Update

- The iterative optimization loop begins. The algorithm formally attempts to update particle positions (P) using the equation: $P_{new} = P_{old} + V$.

- Given that the initial velocities (V) are zero, the probabilities remain static during the first iteration, anchoring the particles at their randomly initialized coordinates.

3. Feature Subset Generation via Binary Thresholding

- To interface with machine learning estimators requiring discrete feature selections, continuous probabilities are discretized.
- For each probability value within the matrix, a random threshold $t \in [0, 1]$ is generated.
- A binary activation is applied: if the particle's probability for a specific feature is $\geq t$, the feature is included (1). Conversely, if the probability is $< t$, the feature is excluded (0).

4. Performance Evaluation

- The designated machine learning estimator (e.g., LASSO Regressor) is trained utilizing exclusively the feature subset generated in the previous step.
- Model efficacy is evaluated using K-Fold cross-validation.
- The mean cross-validation score (e.g., negative mean squared error) is calculated and assigned to the respective particle, serving as its fitness scalar for the current iteration.

5. Global Best Update

- The algorithm compares all fitness scores across the swarm for the current iteration.
- If the maximal score transcends the historical global_best, the global records are updated: global_best adopts the new highest cross-validation score, and global_best_particle adopts the exact probability vector responsible for generating the optimal subset.

6. Velocity Calculation

- Diverging from the standard PSO, this implementation employs a simple, iteration-dependent velocity calculation:

$$v = (gBest - P) / I_{remain}$$

- Where $gBest$ is the optimal probability vector, P is the current probability vector, and I_{remain} denotes the remaining iterations. This formulation calculates the exact difference between a particle and the global leader, divided by the remaining iteration number. Consequently, the fractional step toward the global-best vector increases as the number of remaining iterations decreases.

7. Position Update

• The velocity is applied to the current position matrix to prepare for the subsequent iteration:

$$P_{new} = P_{old} + V$$

• This shift forces the feature-selection probabilities to converge toward the vector that yielded the optimal model performance.

8. Iterative Optimization Loop

• The process from step 3 to step 7 repeats recursively.

• With each iteration, I_{remain} decreases, triggering new stochastic thresholds, model retraining, potential global best updates, and accelerating particle trajectories toward the optimal feature space.

9. Convergence and Final Output (Max Iteration)

• The loop terminates when I_{remain} = 0.

• The algorithm outputs the final convergence metrics: the indices of the optimal feature subset, and the converged probability vector that generated the winning subset.

Table S-3 Characteristics of clinical variables for discovery and holdout test and external validation datasets.

Variable	Discovery Dataset<br><br>Median (range)	Holdout Test Dataset<br><br>Median (range)	Statistical Cohort Comparison	Discovery Dataset<br><br>Median (range)	External Validation Dataset<br><br>Median (range)	Statistical Cohort Comparison
Age (Years)	62.4<br><br>[19.0-86.7]	60.6<br><br>[27.8-85.9]	U: 0.55,<br><br>p-value: 0.3	62.4<br><br>[19.0-86.7]	64.0<br><br>[45.0-78.0]	U: 0.46,<br><br>p-value: 0.55
OS (months)	12.05<br><br>[0.17-52.03]	14.44<br><br>[1.0-58.9]	U: 0.47,<br><br>p-value: 0.58	12.05<br><br>[0.17-52.03]	12.13<br><br>[3.0-41.47]	U: 0.47,<br><br>p-value: 0.59

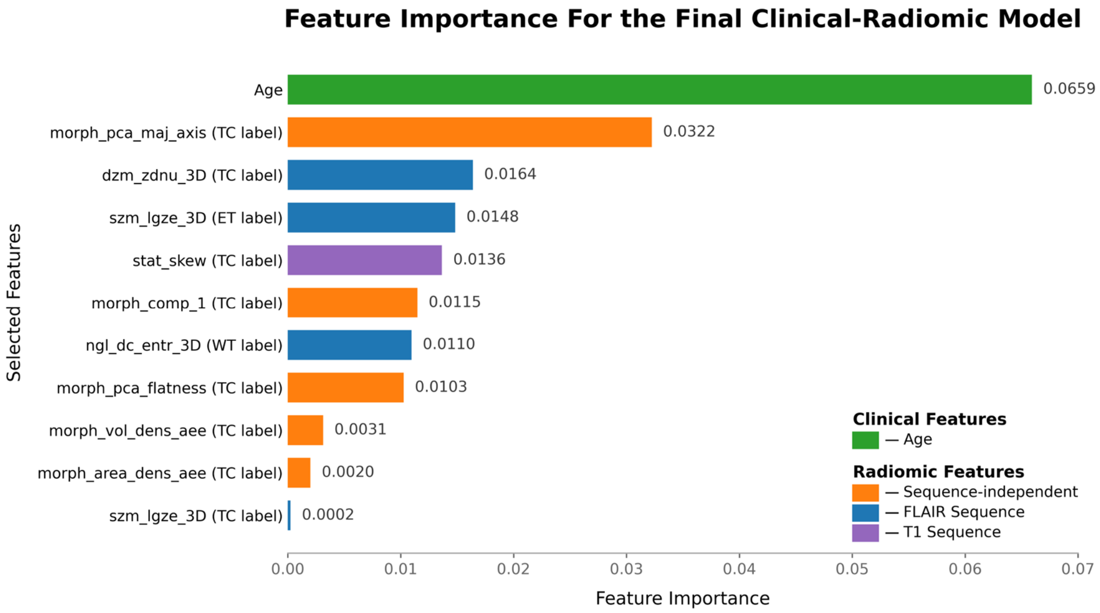

Figure S-4 Feature importance for the final clinical-radiomic model.

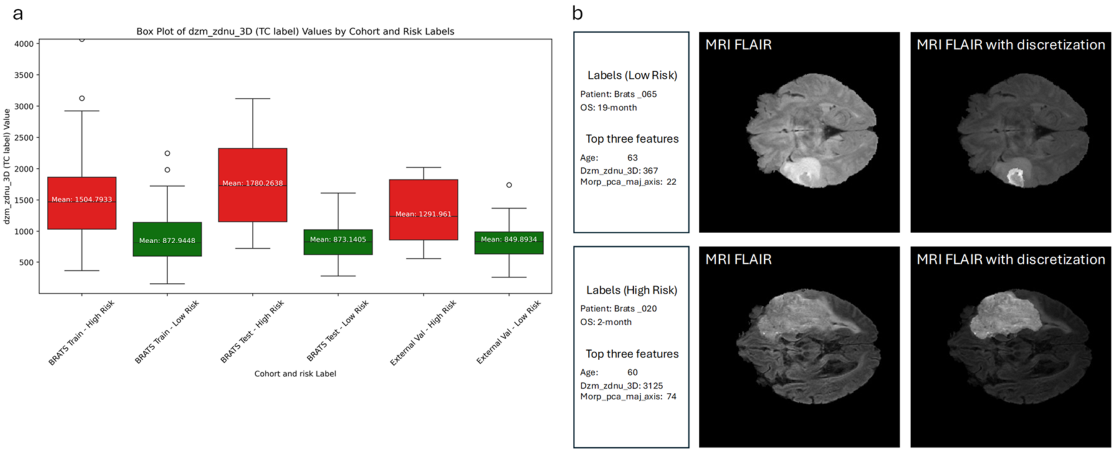

Figure S-5 Feature-level model interpretation using Dzm_zdnu_3D (FLAIR sequence, TC label). (a) Box plots illustrating the stratification of risk groups across the discovery, holdout test, and external validation cohorts. (b) Patient-level analysis of the discovery cohort. Data representation includes Overall Survival (OS) alongside the top three features from the final clinical-radiomic model. RFs were extracted from MRI FLAIR scans, shown with and without discretization.

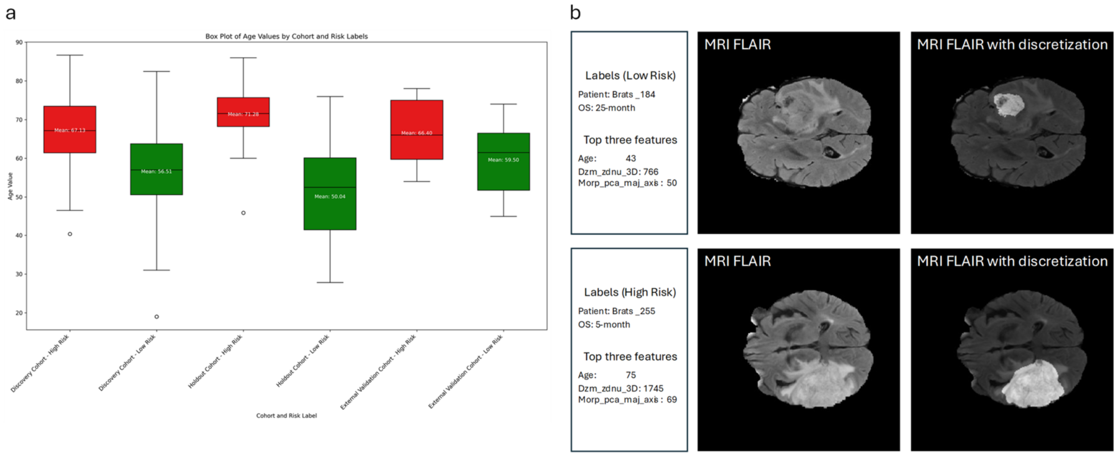

Figure S-6 Feature-level model interpretation using Age. (a) Box plots illustrating the stratification of risk groups across the discovery, holdout test, and external validation cohorts. (b) Patient-level analysis of the discovery cohort. Data representation includes Overall Survival (OS) alongside the top three features from the final clinical-radiomic model. RFs were extracted from MRI FLAIR scans, shown with and without discretization

Model	Feature Selection	Hyperparameters<br><br>(selected [min, max])
Cox-LASSO	LASSO-RANK	alpha = 150 [50,100,150,200]
Cox-LASSO	LASSO-GA	alpha = 200 [50,100,150,200]
Cox-LASSO	LASSO-PSO	alpha = 50 [50,100,150,200]]
RSF	LASSO-RANK	n_estimators = 10 [3,10] max_depth = 5 [3,5] min_samples_split =3 [3,10]
RSF	LASSO-GA	n_estimators = 10 [3,10] max_depth = 5 [3,5] min_samples_split =3 [3,10]
RSF	LASSO-PSO	n_estimators = 10 [3,10] max_depth = 4 [3,5] min_samples_split =10 [3,10]

Table S-4 Hyperparameters for each model and feature selection method from 200 bootstrapped iterations of the training (discovery) dataset.

Feature Selection Method	n_feature pools	Discovery Cohort (C-Index)	Holdout Test Cohort (C-Index	Feature Number (Including Age)
LASSO-RANK	2	0.60	0.63	3
	3	0.59	0.62	4
	4	0.59	0.59	5
	**5**	**0.57**	**0.65**	**6**
	6	0.62	0.58	7
	7	0.62	0.58	8
	8	0.63	0.58	9
	9	0.62	0.60	10
	n_feature pools	Discovery Cohort (C-Index)	Holdout Test Cohort (C-Index	Feature Number (Including Age)
LASSO-PSO	2	0.62	0.58	6
	3	0.64	0.55	7
	4	0.64	0.55	7
	5	0.64	0.57	12
	6	0.65	0.58	10
	7	0.65	0.55	10
	8	0.65	0.58	13
	**9**	**0.64**	**0.62**	**11**
	n_feature pools	Discovery Cohort (C-Index)	Holdout Test Cohort (C-Index	Feature Number (Including Age)
LASSO-GA	2	0.61	0.58	4
	3	0.61	0.58	4
	4	0.61	0.58	4

	5	0.61	0.58	4
	6	0.61	0.58	4
	**7**	**0.62**	**0.59**	**5**
	8	0.61	0.58	4
	9	0.63	0.58	8
	Feature	Discovery Cohort C-index (95% CI)	Holdout Cohort C-index (95% CI)	External Validation Cohort C-index (95% CI)
	Age Only	0.59 (56-64)	0.71 (63-79)	0.60 (49-75)

Table S-5 RFs-only Models and n-feature selection based on the C-index values for holdout dataset. The selected features were used on Cox-LASSO models to check their performance on the discovery, the holdout test. Age-only models are compared to RFs-only models. It generalized poorly on external validation.

LASSO-PSO, Selected Features	Permutation Importance (Feature Importance)	Feature Weight
morph_pca_maj_axis	0.032	0.199
morph_pca_flatness	0.010	0.151
morph_comp_1	0.011	-0.107
morph_vol_dens_aee	0.003	-0.053
morph_area_dens_aee	0.002	0.036
ngl_dc_entr_3D	0.011	-0.137
dzm_zdnu_3D	0.016	0.135
szm_lgze_3D (ET label)	0.015	-0.161
szm_lgze_3D (TC label)	0.0002	-0.007
stat_skew	0.014	-0.132
Age	0.066	0.308
KM curve cut-off value	0.0096 (median)	

Table S-6 Feature importance and weights of each feature in the final clinical-radiomic mode.

Top three features	Low-Risk Criteria	High-Risk Criteria
Age	<57	>67
Morph_pca_maj_axis	<43	>54
Dzm_zdnu_3D	<873	>1504

Table S-7 Dual thresholds of top three features from the final model.

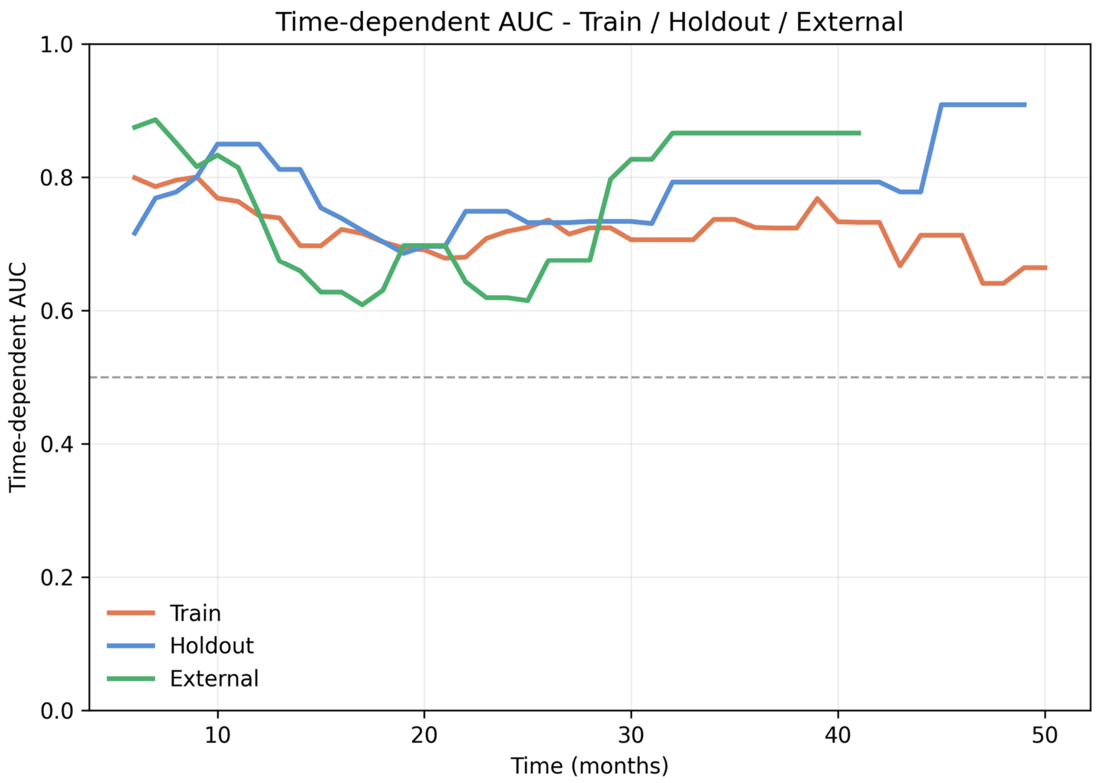

Figure S-7 Time-dependent AUC for Train (Discovery), Holdout, External cohorts.

a

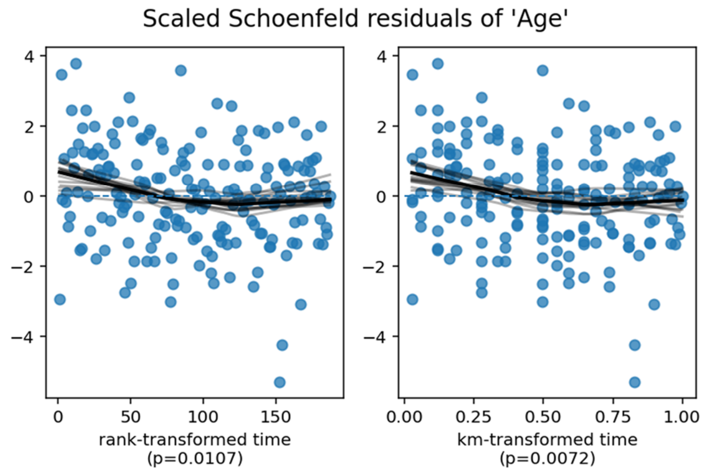

b

Statistical Test Analysis Report

Summary of Kaplan-Meier (km) and log-rank/stratified tests across various imaging features and age.

Feature / Variable	Method Type	Test Statistic	p-value	-log2(p)
Age	km	7.23	**0.01**	7.12
Age	rank	6.51	**0.01**	6.54
flair_et_szm_lgze_3D	km	3.04	0.08	3.62
flair_et_szm_lgze_3D	rank	3.09	0.08	3.67
flair_tc_dzm_zdnu_3D	km	0.01	0.94	0.09
flair_tc_dzm_zdnu_3D	rank	0.00	0.97	0.04
flair_tc_szm_lgze_3D	km	2.06	0.15	2.73
flair_tc_szm_lgze_3D	rank	2.04	0.15	2.70
flair_wt_ngl_dc_entr_3D	km	1.31	0.25	1.99
flair_wt_ngl_dc_entr_3D	rank	1.31	0.25	1.98
t1_tc_stat_skew	km	0.37	0.54	0.88
t1_tc_stat_skew	rank	0.32	0.57	0.81
t2_tc_morph_area_dens_aee	km	0.28	0.59	0.75
t2_tc_morph_area_dens_aee	rank	0.24	0.62	0.68
t2_tc_morph_comp_1	km	1.06	0.30	1.72
t2_tc_morph_comp_1	rank	1.00	0.32	1.66
t2_tc_morph_pca_flatness	km	0.85	0.36	1.49
t2_tc_morph_pca_flatness	rank	0.94	0.33	1.59
t2_tc_morph_pca_maj_axis	km	1.84	0.18	2.51
t2_tc_morph_pca_maj_axis	rank	1.75	0.19	2.43
t2_tc_morph_vol_dens_aee	km	2.21	0.14	2.86
t2_tc_morph_vol_dens_aee	rank	2.11	0.15	2.77

Figure S-8 Proportional hazard assumptions (Scaled Schoenfeld): a) the plot of Age feature (0.01<0.05). b) each RFs and age in the cox model.
